# Supplementary material for: Multivalent binding of the tardigrade Dsup protein to chromatin promotes yeast survival and longevity upon exposure to oxidative damage
Source: Nat Commun. 2025 Sep 29;16:8617. doi: 10.1038/s41467-025-63652-3 (PMC12480509; doi:10.1038/s41467-025-63652-3)

SUPPLEMENTARY MATERIAL

Multivalent binding of the tardigrade Dsup protein to chromatin promotes yeast survival and longevity upon exposure to oxidative damage

Rhiannon R. Aguilar[1,2,*], Laiba F. Khan[3,*], Christopher K. Cummings[4], Nina Arslanovic[1], Thea Grauer[1], Kaylah Birmingham[1,5], Kritika Kasliwal[1,6], Spike D.L. Posnikoff[1], Ujani Chakraborty[1], Allison R. Hickman[3], Rachel Watson[3], Ryan J. Ezell[3], Sabrina R. Hunt[3], Laylo Mukhsinova[3], Hannah E. Willis[3], Martis W. Cowles[3], Richard Garner[1,6], Abraham Shim[1,6], Ignacio Gutierrez[1], Bryan J. Venters[3], Matthew R. Marunde[3], Brian D. Strahl[4], Michael-Christopher Keogh[3,†, +] and Jessica K. Tyler[1,†, +]

[*] These authors contributed equally

[†] Correspondence: mkeogh@epicypher.com and jet2021@med.cornell.edu

[+] These authors jointly supervised this work

[1] Department of Pathology and Laboratory Medicine, Weill Cornell Medicine, New York, NY 10065, USA

[2] Weill Cornell / Rockefeller / Sloan-Kettering Tri-Institutional MD-PhD Program, New York, NY 10065, USA

[3] *EpiCypher Inc*., Durham, NC 27709, USA

[4] Department of Biochemistry and Biophysics and Lineberger Comprehensive Cancer Center, University of North Carolina at Chapel Hill, Chapel Hill, NC 27599, USA

[5] Pharmacology Graduate Program, Weill Cornell Medicine, New York, NY 10065 USA

[6] Biochemistry, Cellular, and Molecular Biology Graduate Program, Weill Cornell Medicine, New York, NY 10065, USA.

SUPPLEMENTARY DATA FILES

Supplementary Data File 1A-E: Resources

Tab A: Primer sequences

Tab B: Yeast strains (and their phenotypes)

Tab C: CUT&RUN antibodies

Tab D: Captify Targets (nucleosomes and free DNA)

Tab E: Captify Queries (+ Misc protein info)

Supplementary Data File 2: CUT&RUN sequence stats. [Related to **Fig. 4b**]

Supplementary Data File 3: RNA-seq results for the 868 genes significantly differentially expressed between any condition (yeast +/- Dsup exposed to H_2O_2 (0, 4 or 8mM) for 30 mins; T0 is untreated). Data shown are the average of triplicate experiments. [Related to **Fig. 5**]

Supplementary Data File 4: Compiled Captify data. [Related to noted **Figs**]

Tab 1. Fig. 6a: Dsup (WT) Titration (0 µg/mL SalDNA) - 150 mM & 250 mM NaCl

Tab 2. Fig. 6b: Dsup (WT) Titration ± (1 µg/mL SalDNA)

Tab 3. Fig. 6c: Dsup (HMGN-3R/3E, HMGN-8A, Dsup ΔC) Titration ± (1 µg/mL SalDNA)

Tab 4. Fig. 8a: Dsup (WT) Titration w/ tail truncated Nucs (1 µg/mL SalDNA)

Tab 5. Fig. 8b: Dsup (WT) Titration w/ select acyl Nucs (1 µg/mL SalDNA)

Tab 6. Fig. 8c: Dsup (WT) Titration w/ select methyl Nucs (1 µg/mL SalDNA)

Tab 7. Fig. 8d: Dsup (WT) Titration w/ acidic patch mutant Nucs (1 µg/mL SalDNA)

Tab 8. Supplementary Fig. 8a: Dsup (WT) Titration (± 0.74 µg/mL SalDNA)

Tab 9. Supplementary Fig. 8b: Dsup (ΔHMGN ΔC) Titration (± 0.74 µg/mL SalDNA)

Tab 10. EC_{50}^{rel} Table for all Captify figures

SUPPLEMENTARY FIGURE LEGENDS

Supplementary Fig. 1. Impact of Dsup on yeast chronological and replicative lifespan. **a.** In otherwise WT yeast Dsup had negligible impact on chronological lifespan (the length of time a cell survives in a non-dividing state). EV, Empty vector. **b.** Dsup expression slightly reduces yeast replicative lifespan (the maximum number of times a cell can divide). All strain genotypes in **Supplementary Data File 1B**. Source data is provided in the Source Data file.

Supplementary Fig. 2. Cell compartmentalization of redox reporters. roGFP-Grx1 (redox sensitive GFP - GlutaRedoXin 1) localizes to the cytoplasm (left), while an NLS fusion (roGFP-Grx1-NLS) localizes to the nucleus (right).

Supplementary Fig. 3. Micrococcal Nuclease (MNase) digestion of yeast chromatin. **a-b.** Isolated chromatin from crosslinked yeast cells containing Dsup (WT) or Empty vector (EV) was untreated (Input) or incubated with MNase (times indicated), purified to DNA, and resolved by agarose gel electrophoresis (see Methods). MW, DNA molecular weight standards. *, **, *** : mono-, di-, or tri- nucleosome sized DNA fragments. Source data is provided in the Source Data file.

Supplementary Fig. 4. CUT&RUN DNA yields from three independent analyses of yeast strains containing Empty vector (EV), Dsup (WT), Dsup ΔHMGN ΔC +NLS or Dsup HMGN-3R/3E (6His-Dsup-FLAG: each enriched with anti-FLAG). Controls are IgG and anti-H3K4me3. All antibodies in **Supplementary Data File 1C**. Source data is provided in the Source Data file.

Supplementary Fig. 5. Gcn4 OX (overexpressed) has elevated levels of the transcriptional activator, not observed in the presence of Dsup (WT or mutants). Immunoblot of total protein

extracts from indicated strains (**Supplementary Data File 1B**) detected by anti-Gcn4 or anti-H3 (loading control) of samples ran on the same gel. Source data is provided in the Source Data file.

Supplementary Fig. 6. Purified Dsup proteins (format: 6His-Dsup-FLAG) used for Captify analyses (*e.g.*, **Figs. 6** and **8** and **Supplementary Fig. 8**). Recombinant proteins were purified, resolved by 15% SDS-PAGE and stained with Coomassie Brilliant Blue dye (see Methods). Source data is provided in the Source Data file.

Supplementary Fig. 7. Schematic of Captify-Alpha assay to measure the interaction between epitope-tagged Dsup Queries (**Supplementary Data File 1E**) and biotinylated nucleosome or 147x601 free DNA Targets (green circle represents biotin bound to the streptavidin Donor bead). Donor bead are laser excited (680 nM), causing release of a short-lived singlet oxygen molecule, and emission (520-620 nM) from proximal (within 200 nm) anti-tag Acceptor beads; this luminescent signal is directly proportional to the amount of [Donor-Acceptor] bridged by the [Target : Query] interaction. To compare across each [Target : Query], data is presented as their relative concentration effective in producing 50% of the maximal response (EC_{50}^{rel}) by plotting Alpha Counts (fluorescence) as a function of protein concentration[1]. The assay can be performed under a variety of conditions (*e.g.*, ionic strength) or -/+ modulators (*e.g.*, competitor salmon sperm DNA (salDNA)). For representative data see **Figs. 6** and **8** and **Supplementary Fig. 8. Supplementary Data File 4** contains all EC_{50}^{rel} from this study.

Supplementary Fig. 8. Complete deletion of the Dsup HMGN and adjacent C-terminal deletion (ΔHMGN ΔC; Δ aa 359-455) ablates nucleosome and DNA binding. **a-b**. Captify assay using 6His-Dsup-FLAG queries (concentrations on X-axis; WT (**a**) or mutant (**b**) as noted below) and biotinylated 147x601 (free) DNA, unmodified nucleosome (rNuc) or tail-less nucleosome (trypsin

digested as in **Fig. 8a**) targets. All assays performed under optimized conditions (from **Fig. 6b**): Dsup (WT or mutant in 1.5-fold serial dilutions), nucleosome (each as indicated; 10 nM), free DNA (147x601; 2.5 nM), 150 mM NaCl, 0 or 0.74 (-/+) μg/ml salDNA competitor. Source data is provided in the Source Data file.

Supplementary References

1 Marunde, M. R., Popova, I. K., Weinzapfel, E. N. & Keogh, M. C. The dCypher Approach to Interrogate Chromatin Reader Activity Against Posttranslational Modification-Defined Histone Peptides and Nucleosomes. *Methods Mol Biol* **2458**, 231-255, doi:10.1007/978-1-0716-2140-0_13 (2022).

Supplementary Fig. 1

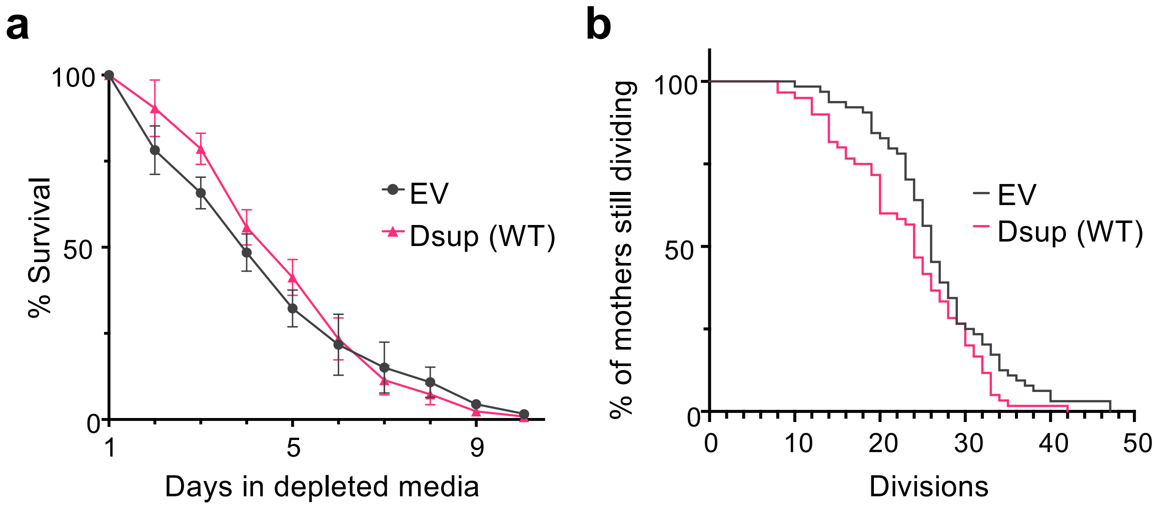

Supplementary Fig. 2

roGFP2-Grx1 roGFP-Grx1_NLS

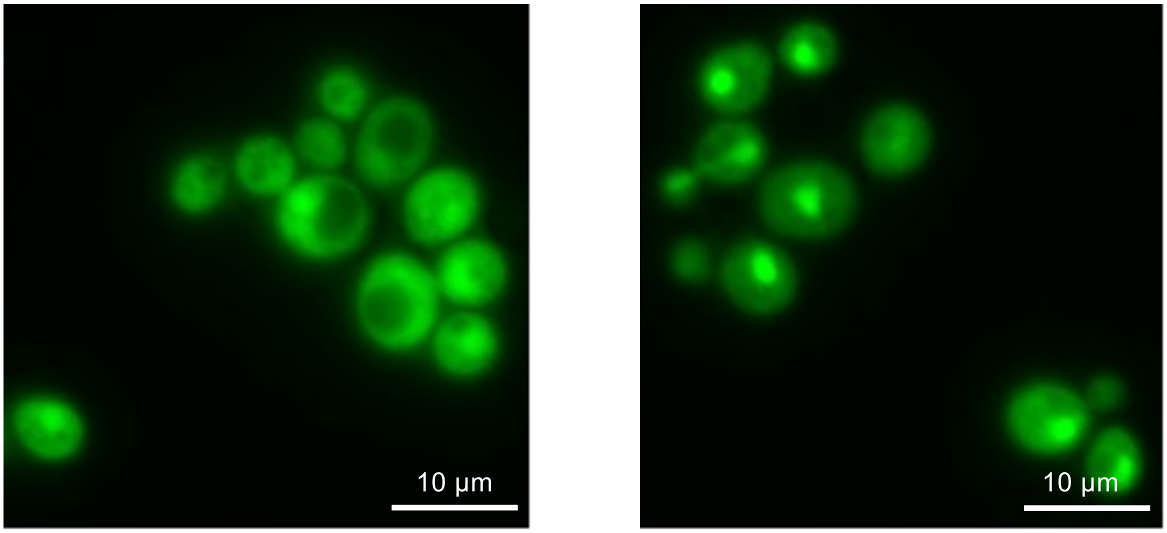

Supplementary Fig. 3

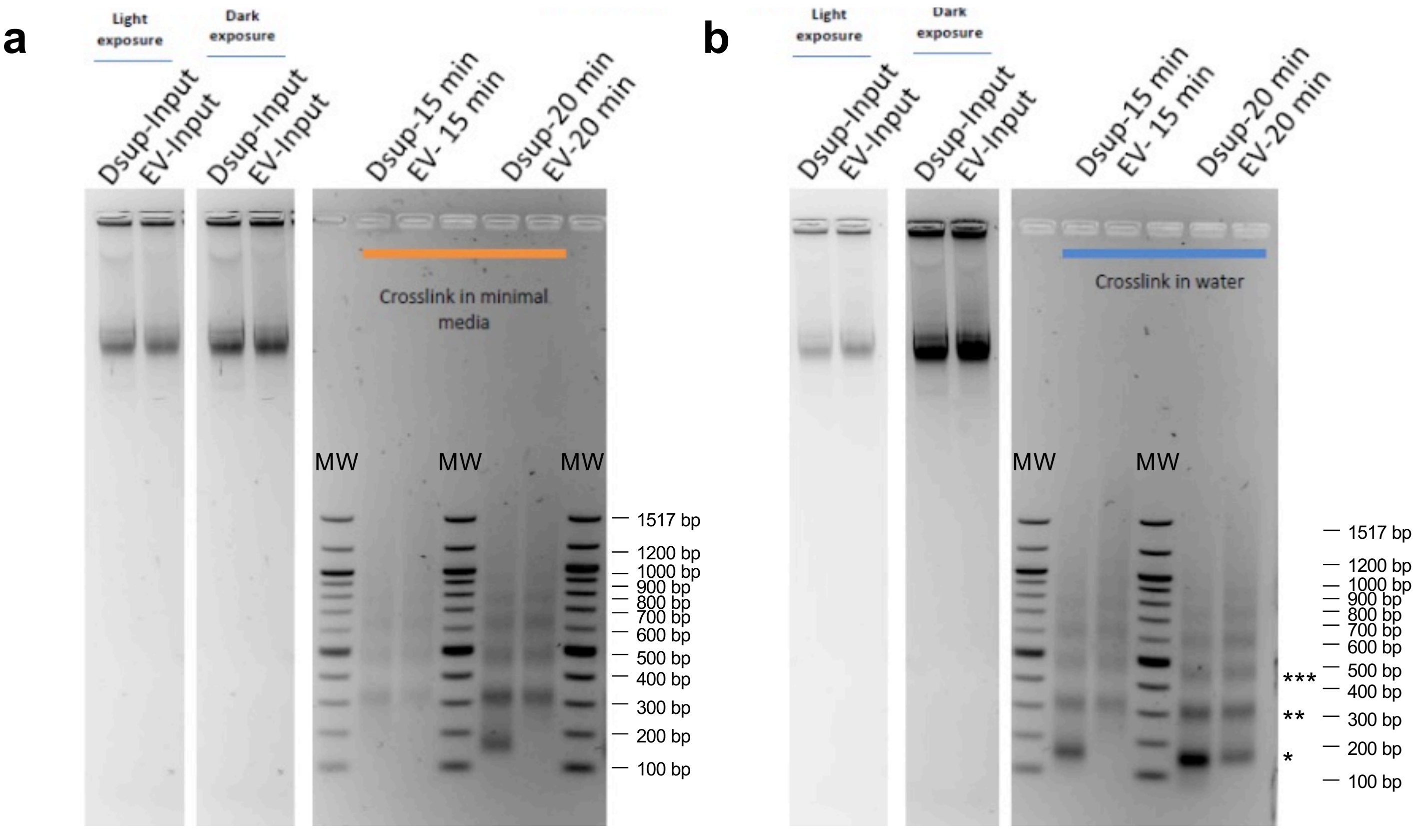

Supplementary Fig. 4

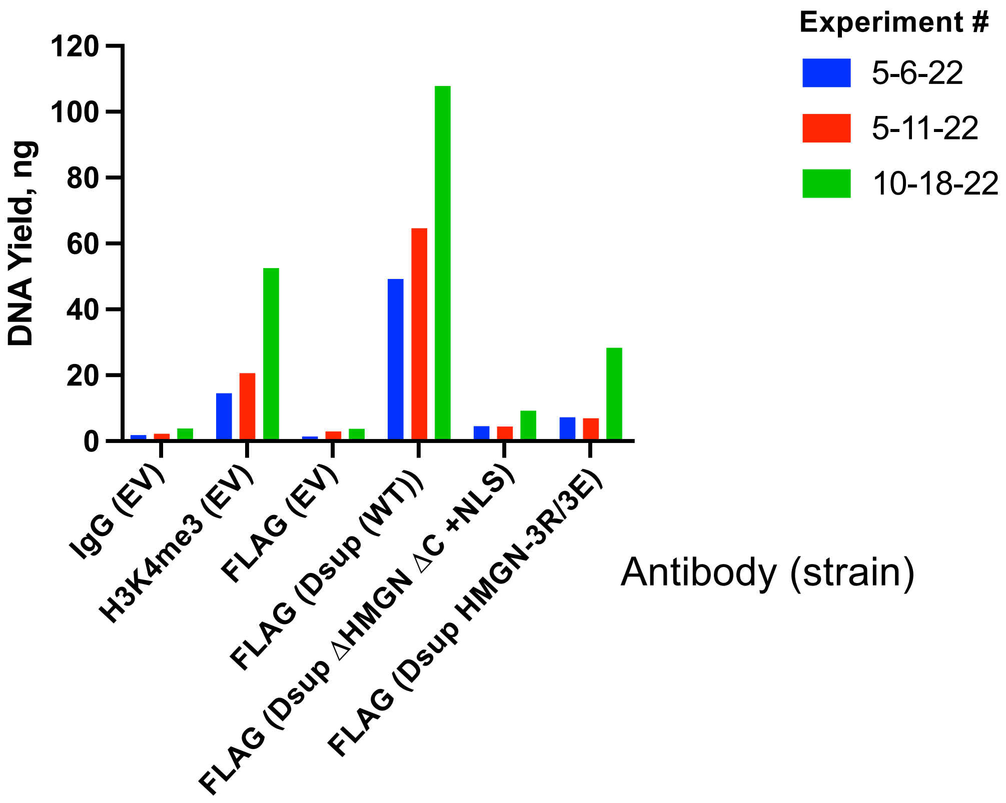

Supplementary Fig. 5

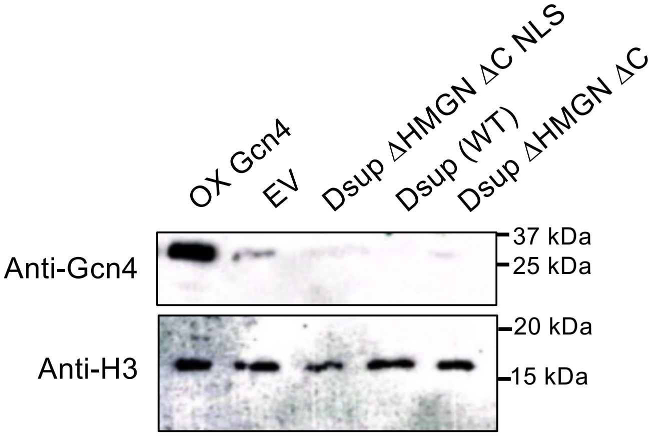

Supplementary Fig. 6

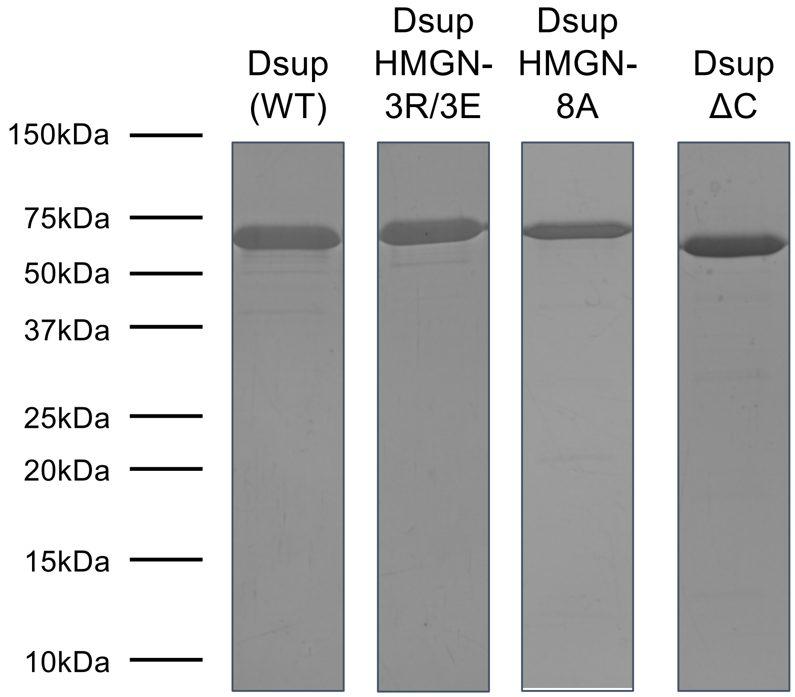

Supplementary Fig. 7

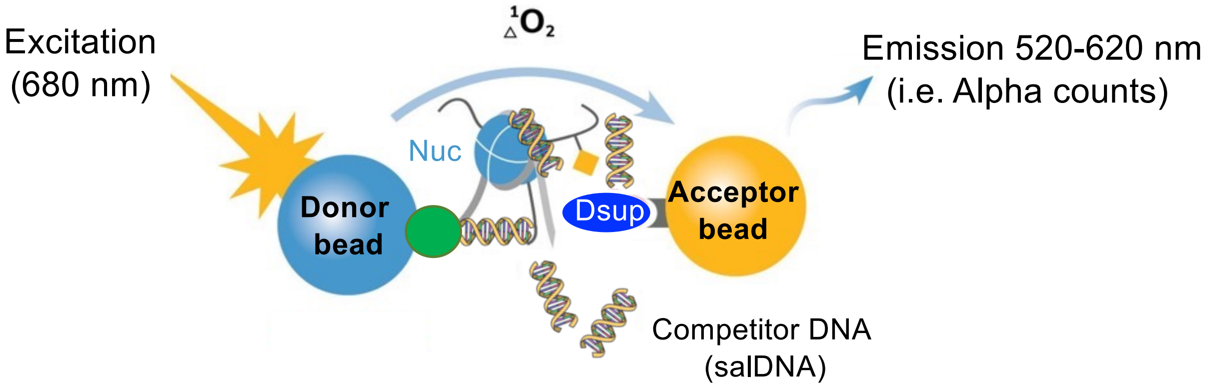

Supplementary Fig. 8

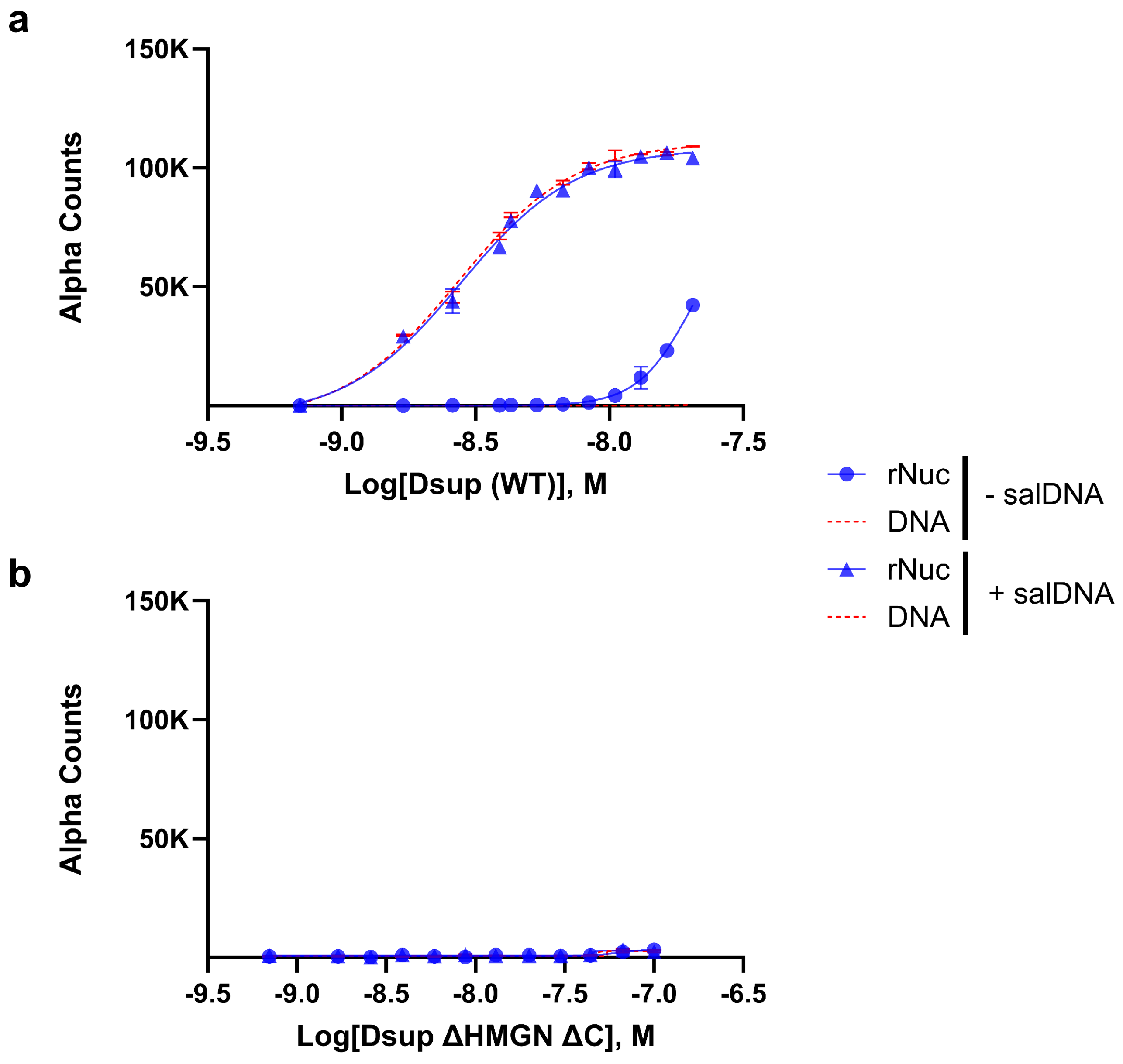

Supplement: Supplementary file 1 — Supplementary Information [file 41467_2025_63652_MOESM1_ESM.pdf]
