# Supplementary material for: Multivalent binding of the tardigrade Dsup protein to chromatin promotes yeast survival and longevity upon exposure to oxidative damage
Source: Nat Commun. 2025 Sep 29;16:8617. doi: 10.1038/s41467-025-63652-3 (PMC12480509; doi:10.1038/s41467-025-63652-3)
Supplement: Supplementary file 3 — Description of Additional Supplementary Files [file 41467_2025_63652_MOESM3_ESM.pdf]

Description of Additional Supplementary Files

File Name: Supplementary Data 1
Description: Resources list

File Name: Supplementary Data 2
Description: CUT and RUN sequencing stats

File Name: Supplementary Data 3
Description: RNAseq data

File Name: Supplementary Data 4
Description: Captive Data
