# Supplementary material for: Multivalent binding of the tardigrade Dsup protein to chromatin promotes yeast survival and longevity upon exposure to oxidative damage
Source: Nat Commun. 2025 Sep 29;16:8617. doi: 10.1038/s41467-025-63652-3 (PMC12480509; doi:10.1038/s41467-025-63652-3)
Supplement: Supplementary file 8 — Reporting Summary [file 41467_2025_63652_MOESM8_ESM.pdf]

nature portfolio

Corresponding author(s): Michael-Christopher Keogh (mkeogh@epicypher.com) & **Jessica K. Tyler (jet2021@med.cornell.edu)**

Last updated by author(s): July 24th 2025

Reporting Summary

Nature Portfolio wishes to improve the reproducibility of the work that we publish. This form provides structure for consistency and transparency in reporting. For further information on Nature Portfolio policies, see our Editorial Policies and the Editorial Policy Checklist.

Please do not complete any field with "not applicable" or n/a. Refer to the help text for what text to use if an item is not relevant to your study.
For final submission: please carefully check your responses for accuracy; you will not be able to make changes later.

Statistics

For all statistical analyses, confirm that the following items are present in the figure legend, table legend, main text, or Methods section.

n/a	Confirmed	
☐	☒	The exact sample size (*n*) for each experimental group/condition, given as a discrete number and unit of measurement
☐	☒	A statement on whether measurements were taken from distinct samples or whether the same sample was measured repeatedly
☐	☒	The statistical test(s) used AND whether they are one- or two-sided<br>*Only common tests should be described solely by name; describe more complex techniques in the Methods section.*
☒	☐	A description of all covariates tested
☒	☐	A description of any assumptions or corrections, such as tests of normality and adjustment for multiple comparisons
☐	☒	A full description of the statistical parameters including central tendency (e.g. means) or other basic estimates (e.g. regression coefficient) AND variation (e.g. standard deviation) or associated estimates of uncertainty (e.g. confidence intervals)
	☒	For null hypothesis testing, the test statistic (e.g. *F*, *t*, *r*) with confidence intervals, effect sizes, degrees of freedom and *P* value noted<br>*Give P values as exact values whenever suitable.*
☒	☐	For Bayesian analysis, information on the choice of priors and Markov chain Monte Carlo settings
☒	☐	For hierarchical and complex designs, identification of the appropriate level for tests and full reporting of outcomes
☒	☐	Estimates of effect sizes (e.g. Cohen's *d*, Pearson's *r*), indicating how they were calculated

Our web collection on statistics for biologists contains articles on many of the points above.

Software and code

Policy information about availability of computer code

Data collection	See below (abridged due to box size restriction / full details in methods)
Data analysis	**Replicative lifespan analysis:** At least 50 cells were counted per condition, with survival curves calculated on Graphpad Prism 9, and statistical analysis performed with a log-rank test.<br><br>**Redox analysis:** The mean of the 405/488 nm values for each timepoint was calculated using FlowJo. Data is presented as the mean and standard deviation of three independent cultures and compared using a student's t-test.<br><br>**CUT&RUN Analysis:** CUT&RUN studies were performed independently three times with consistent results. Full details on analysis (including all software packages) as in methods. Bowtie2, BEDTools v2.30.0<br><br>**RNA-seq Analysis:** For each biological replicate, three technical replicates were prepared from mRNA and sequenced. Full details on analysis (including all software packages) as in methods. FastQC v0.11.9, Bowtie2, Homer v4.11, EdgeR v3.34.0, DAVID v2024q2<br><br>**dCypher analysis:** Binding curves were plotted in GraphPad Prism 9.0 using 4-parameter logistic nonlinear regression.

For manuscripts utilizing custom algorithms or software that are central to the research but not yet described in published literature, software must be made available to editors and reviewers. We strongly encourage code deposition in a community repository (e.g. GitHub). See the Nature Portfolio guidelines for submitting code & software for further information.

Data

Policy information about availability of data

All manuscripts must include a data availability statement. This statement should provide the following information, where applicable:

- Accession codes, unique identifiers, or web links for publicly available datasets
- A description of any restrictions on data availability
- For clinical datasets or third party data, please ensure that the statement adheres to our policy

CUT&RUN sequence data is publicly available from NCBI Gene Expression Omnibus at accession number **GSE237436** [ncbi.nlm.nih.gov/geo/query/acc.cgi?acc=GSE237436]. RNA-sequence data is publicly available from NCBI Gene Expression Omnibus at accession number **GSE294109** [ncbi.nlm.nih.gov/geo/query/acc.cgi?acc=GSE294019].

Research involving human participants, their data, or biological material

Policy information about studies with human participants or human data. See also policy information about sex, gender (identity/presentation), and sexual orientation and race, ethnicity and racism.

Reporting on sex and gender	Not Relevant
Reporting on race, ethnicity, or other socially relevant groupings	Not Relevant
Population characteristics	Not Relevant
Recruitment	Not Relevant
Ethics oversight	Not Relevant

Note that full information on the approval of the study protocol must also be provided in the manuscript.

Field-specific reporting

Please select the one below that is the best fit for your research. If you are not sure, read the appropriate sections before making your selection.

☒ Life sciences ☐ Behavioural & social sciences ☐ Ecological, evolutionary & environmental sciences

For a reference copy of the document with all sections, see nature.com/documents/nr-reporting-summary-flat.pdf

Life sciences study design

All studies must disclose on these points even when the disclosure is negative.

Sample size	Sample sizes for each technique as standard for each type of analysis, from the literature. No specific tests were used.
Data exclusions	No exclusions
Replication	Independent runs of each approach (with appropriate controls) were performed and a representative shown All attempts at replication were successful
Randomization	Samples allocated to groups by Dsup allele status (for relevant in vivo / in vitro analyses)
Blinding	Samples not blinded (not a standard approach for field / techniques used)

Reporting for specific materials, systems and methods

We require information from authors about some types of materials, experimental systems and methods used in many studies. Here, indicate whether each material, system or method listed is relevant to your study. If you are not sure if a list item applies to your research, read the appropriate section before selecting a response.

Materials & experimental systems

n/a	Involved in the study
☐	☒ Antibodies
☒	☐ Eukaryotic cell lines
☒	☐ Palaeontology and archaeology
☐	☒ Animals and other organisms
☒	☐ Clinical data
☒	☐ Dual use research of concern
☒	☐ Plants

Methods

n/a	Involved in the study
☐	☒ ChIP-seq
☒	☐ Flow cytometry
☒	☐ MRI-based neuroimaging

Antibodies

Antibodies used

Immunoblot : anti-FLAG (Sigma F1804, 1:1,000) and anti-GAPDH (Sigma A9521, 1:10,000)

Chromatin Fractionation / Immunoblot: anti-FLAG (Sigma F1804, 1:1,000), anti-H2A (Abcam ab18255, 1:5,000), anti-GAPDH (Sigma A9521, 1:20,000)

CUT&RUN: see Supplementary Table 1 / Resources C and GSE237436
IgG EpiCypher #13-0042 (Lot 20036001-52)
H3K4me3, SNAP-Certified EpiCypher # 13-0041 (Lot SG2419844A)
FLAG ThermoFisher #MA1-91878 (Lot 19091001)

Target protein	Supplier	Catalog Number	Clone name	Lot number	Dilution
FLAG	Sigma	F1804	**M1**	0000278731	1 in 1000
GAPDH	Sigma	A9521	**Polyclonal**	71K9260	1 in 10,000
H2B	Abcam	ab1790	**Polyclonal**	1079078-14	1 in 5,000
H2A	Active Motif	39235	**Polyclonal**	12819001	1 in 2000
GCN4	Absolute Antibody	Ab00436-1.1	C11L34	T1541A11	1 in 1000
H3	Abcam	ab1791	**Polyclonal**	1015776-1	1 in 1000
Anti-Mouse IgG HRP Conjugate	Promega	W402B	N/A	421603	1 in 2500
Anti-Rabbit IgG HRP Conjugate	Promega	W401B	N/A	417842	1 in 5000

Immunofluoresence:
Primary antibodies: anti-H2A (Abcam ab18255, 1:1.000), anti-GAPDH (Sigma A9521, 1:5,000), or anti-FLAG (Sigma F1804, 1:1,000)
Secondary antibodies: Alexa Fluor® 594 or 488 as noted (BioLegend)

Validation

All antibodies had accompanying supplier validation. Those for CUT&RUN are further validated at EpiCypher to in-assay spike-in nucleosomes. Antibodies were validated for western by use of strains with deletions of the relevant gene, or lacking the epitope tag.

Animals and other research organisms

Policy information about studies involving animals; ARRIVE guidelines recommended for reporting animal research, and Sex and Gender in Research

Laboratory animals: For all Yeast Strains see [Suppl. Table 1A-E: Resources Tab B]

Wild animals: Not Relevant

Reporting on sex: Not Relevant

Field-collected samples: Not Relevant

Ethics oversight: Not Relevant

Note that full information on the approval of the study protocol must also be provided in the manuscript.

Plants

Seed stocks: Not Relevant

Novel plant genotypes: Not Relevant

Authentication: Not Relevant

ChIP-seq

Data deposition

☒ Confirm that both raw and final processed data have been deposited in a public database such as GEO.

☒ Confirm that you have deposited or provided access to graph files (e.g. BED files) for the called peaks.

Data access links
May remain private before publication.

CUT&RUN: All sequencing data is publicly available at the NCBI Gene Expression Omnibus (GEO) with accession number GSE237436. [https://www.ncbi.nlm.nih.gov/geo/query/acc.cgi?acc=GSE237436]

Files in database submission

RAW FILES
file name file checksum
EmptyVector_IgG_R1_001.fastq.gz 84fbe5c985e0d97cc2d6519458f3ee7c
EmptyVector_IgG_R2_001.fastq.gz a62cad8b798c5b7d461563b1b84fb3d2
Dsup_IgG_R1_001.fastq.gz 114febe89f6e79e5d752c70e0c09c869
Dsup_IgG_R2_001.fastq.gz f6b9a60acd5c0b34898f72a02ff13b95
Dsup-M1-dCNLS_IgG_R1_001.fastq.gz c96221b2b151826b24d8059d21deb237
Dsup-M1-dCNLS_IgG_R2_001.fastq.gz cde917aa6549c2e7a6a8a70cba60bba3
Dsup-M2-3R3E_IgG_R1_001.fastq.gz 29a3ebd58366713e7e5b14864338c7b2
Dsup-M2-3R3E_IgG_R2_001.fastq.gz c4149d8265201df50becb61c1842d05e
EmptyVector_H3K4me3_R1_001.fastq.gz 453e21a0ccd807f3c9697da34efc939a
EmptyVector_H3K4me3_R2_001.fastq.gz ad835d755aca7ca9d8a21811de227cbf
Dsup_H3K4me3_R1_001.fastq.gz 5c6e57631362ec9bde7d8764ef9e8687
Dsup_H3K4me3_R2_001.fastq.gz 26167416407e8f32e6302260196f6644
Dsup-M1-dCNLS_H3K4me3_R1_001.fastq.gz 5885663f87c60fd1e0dbfe1f5210627a
Dsup-M1-dCNLS_H3K4me3_R2_001.fastq.gz a2f02e87ef8c0b9df079a4951c2aee60
Dsup-M2-3R3E_H3K4me3_R1_001.fastq.gz b252b1ba023e77b197e60d7179b33ac2
Dsup-M2-3R3E_H3K4me3_R2_001.fastq.gz 9d01ad9ab1f9ebf61651c6bc77c884dc
EmptyVector_FLAG_R1_001.fastq.gz 64c5fcf7d10c9cee22aaff2db59e8404
EmptyVector_FLAG_R2_001.fastq.gz edd86a946b49e62734c9d56d7cc89da2
Dsup_FLAG_R1_001.fastq.gz 405a95a0909398e4f651dfa080f6dfa1
Dsup_FLAG_R2_001.fastq.gz 3d5ddafe1fa8d61315aa669be676f9c6
Dsup-M1-dCNLS_FLAG_R1_001.fastq.gz 39d59cea533a644d4a05b820c10cc579
Dsup-M1-dCNLS_FLAG_R2_001.fastq.gz ebf9d68d50ea3e283c287f37a4b23ab8
Dsup-M2-3R3E_FLAG_R1_001.fastq.gz eb58b577f43a36872df08e69dd13117e
Dsup-M2-3R3E_FLAG_R2_001.fastq.gz 352245efdf52884907c884f4acbc54ca

PPROCESSED DATA FILES
file name file checksum
EmptyVector_IgG.eColi.b20s100dt.bw db901a2db7af6156fb907a66f4e96722
Dsup_IgG.eColi.b20s100dt.bw e6ea77d0a3b35f24b9a1efdeaccaad91
Dsup-M1-dCNLS_IgG.eColi.b20s100dt.bw cc3a1f8e0d10b0ae3d68ad72a97eedcd
Dsup-M2-3R3E_IgG.eColi.b20s100dt.bw a80cae0e7d06c058b6b5a0240289cfc9
EmptyVector_H3K4me3.eColi.b20s100dt.bw 1150aed5a456fd9c08b408ad013f42c5
Dsup_H3K4me3.eColi.b20s100dt.bw 6d0b9bada4e8a2c6f1f0973d5fb08be2
Dsup-M1-dCNLS_H3K4me3.eColi.b20s100dt.bw 9654871dec6f2988d06c5b653ceb1f60
Dsup-M2-3R3E_H3K4me3.eColi.b20s100dt.bw 937f7d809a5dac24f2762101b28f6194
EmptyVector_FLAG.eColi.b20s100dt.bw 95d6376c8497b3065cf8b38088d5ed5e
Dsup_FLAG.eColi.b20s100dt.bw 9ae066a7b81e3fa00fd24ddf20ea31a3
Dsup-M1-dCNLS_FLAG.eColi.b20s100dt.bw b44ec5a6697d458feb2428c5ca170687
Dsup-M2-3R3E_FLAG.eColi.b20s100dt.bw f1a3a6860920a1d366294c4875dc4cf4

Genome browser session
(e.g. UCSC)

Integrative Genomics Viewer (IGV)

Methodology

Replicates

CUT&RUN used Biological Replicates for each condition / target Representative data of three independent experiments shown

Sequencing depth

CUT&RUN ibraries were sequenced on an Illumina NextSeq 2000 platform, obtaining an average of ~1.1 million paired-end reads per reaction (Suppl. Table 2).

Antibodies

See above

Peak calling parameters

Paired-end fastq files were aligned to the sacCer3 reference genome using Bowtie2. Duplicate (SAMtools) and multi- aligned (Picard) reads were filtered, and the resulting unique reads for comparable reactions normalized by an E coli scaling factor (1/ % E coli Reads) (bedtools), and further normalized to RPKM bigwig files (DeepTools). Integrative Genomics Viewer (IGV) was utilized for the visualization of peaks from bigwig files All sequencing data has been deposited in the NCBI Gene Expression Omnibus (GEO) with accession number GSE237436.

Data quality

Passed all standard metrics (Illumina and see above)

Software

Paired-end fastq files were aligned to the sacCer3 reference genome using Bowtie2 (v2.2.5).
Duplicate reads were removed via SAMtools (v1.6).
Multi-aligned reads were removed via Picard (v2.26.2).
bigWig creation included both E. coli normalization and RPKM normalization via bamCoverage in deeptools (v3.5.1). Files were E. coli normalized by an E coli scaling factor (1/%E coli reads) and RPKM normalized (--normalizeUsing option).

is checklist template is licensed under a Creative Commons Attribution 4.0 International License, which permits use, sharing, adaptation, distribution and reproduction in any medium or format, as long as you give ppropriate credit to the original author(s) and the source, provide a link to the Creative Commons license, and indicate if changes were made. The images or other third party material in this article are included in e article's Creative Commons license, unless indicated otherwise in a credit line to the material. If material is not included in the article's Creative Commons license and your intended use is not permitted by atutory regulation or exceeds the permitted use, you will need to obtain permission directly from the copyright holder. To view a copy of this license, visit http://creativecommons.org/licenses/by/4.0/
